# Divergent Prognostic Impact of Germline *BRCA1* and *BRCA2* Variants in Non-Metastatic Breast Cancer: A Real-World Cohort Study

**DOI:** 10.3390/cancers18193073

**Published:** 2026-09-22

**Authors:** Ismail Bayrakci, Rusen Cosar, Necdet Sut, Hakan Gurkan, Omer Faruk Cetinkaya, Eylul Senodeyici, Didem Divriklioglu, Gizem Bakir Kahveci, Ahmet Kucukarda, Muhammet Bekir Hacioglu, Bulent Erdogan, Sernaz Topaloglu

**Affiliations:** 1 Division of Medical Oncology, Department of Internal Medicine, Trakya University School of Medicine, 22130 Edirne, Turkey; 2 Department of Radiation Oncology, Trakya University School of Medicine, 22130 Edirne, Turkey; 3 Department of Biostatistics, Trakya University School of Medicine, 22130 Edirne, Turkey; 4 Department of Medical Genetics, Trakya University School of Medicine, 22130 Edirne, Turkey; 5 Trakya University School of Medicine, 22130 Edirne, Turkey

**Keywords:** breast cancer, non-metastatic breast cancer, *BRCA1*, *BRCA2*, germline pathogenic variant, prognosis, survival analysis

## Abstract

**Background:** Germline pathogenic variants in *BRCA1* and *BRCA2* are established contributors to hereditary breast cancer, yet their separate prognostic effects in non-metastatic disease remain uncertain because they are frequently analyzed as a single group. **Methods:** We retrospectively evaluated 360 patients with stage I–III invasive breast cancer who underwent germline genetic testing. Patients were categorized as *BRCA*-negative (*n* = 281), *BRCA1*-positive (*n* = 43), or *BRCA2*-positive (*n* = 36). Disease-free survival (DFS) and overall survival (OS) were analyzed using Kaplan–Meier methods and Cox proportional hazards models with adjustment for molecular subtype and clinicopathological variables. **Results:** During follow-up, 76 DFS events and 27 deaths were observed. *BRCA2* positivity was independently associated with worse DFS (hazard ratio [HR] 2.13, 95% confidence interval [CI] 1.14–3.98; *p* = 0.017), whereas *BRCA1* positivity showed an association with improved OS (HR 0.08, 95% CI 0.01–0.51; *p* = 0.007). The OS finding should be interpreted cautiously because of the limited number of death events and the risk of model instability. **Conclusions:** Germline *BRCA1* and *BRCA2* variants may have divergent prognostic implications in non-metastatic breast cancer and should not be treated as a single homogeneous prognostic entity. Separate evaluation of these variants may improve risk stratification and support more individualized patient management, although validation in larger cohorts is warranted.

## Simple Summary

Some breast cancers are caused by inherited changes in the *BRCA1* or *BRCA2* genes. These two genes are often discussed together, but they may affect breast cancer behavior in different ways. In this study, we looked at patients with non-metastatic breast cancer who had genetic testing. We compared patients without a *BRCA* variant with patients who had either a *BRCA1* or a *BRCA2* variant. The results suggest that these two genes should not be considered the same when estimating prognosis. Patients with a *BRCA2* variant had a higher risk of the cancer coming back, while patients with a *BRCA1* variant showed better overall survival. However, the survival result should be interpreted carefully because only a small number of deaths occurred. Considering *BRCA1* and *BRCA2* separately may help clinicians better assess patient risk and plan follow-up.

## 1. Introduction

Breast cancer (BC) is the most commonly diagnosed malignancy in women worldwide and remains the second leading cause of cancer-related mortality [1]. Although most BC cases are sporadic, approximately 5–10% are associated with hereditary predisposition. A substantial proportion of hereditary cases are attributable to germline pathogenic variants in the *BRCA1* and *BRCA2* genes, which play essential roles in DNA repair through homologous recombination [2,3]. Individuals carrying pathogenic variants in *BRCA1* or *BRCA2* have a markedly increased lifetime risk of BC, with a considerable proportion of this risk occurring at a younger age [4].

The clinicopathological phenotype of *BRCA*-associated BC differs according to the affected gene. *BRCA1* variants are commonly associated with high-grade, triple-negative, and basal-like tumors [5,6]. In contrast, *BRCA2* variants are more frequently associated with hormone receptor-positive and HER2-negative tumors and often show histological features more similar to sporadic BC [5,7,8]. In particular, luminal subtypes, especially luminal B disease, are commonly observed among *BRCA2* variant carriers, whereas triple-negative disease predominates among *BRCA1* variant carriers. These differences suggest that *BRCA1-* and *BRCA2*-associated tumors may not share the same biological behavior or prognostic profile.

BC is a heterogeneous disease, and molecular subtypes defined by hormone receptor status, HER2 expression, and proliferative activity are central determinants of prognosis [9]. Young age was previously considered an independent adverse prognostic factor; however, more recent evidence suggests that its effect varies according to clinical and molecular subtype [10]. Similarly, although germline *BRCA1* and *BRCA2* variants clearly influence tumor biology, their independent prognostic impact remains uncertain when molecular subtype and other clinicopathological variables are taken into account [5].

In addition to gene-level differences, emerging evidence indicates that the type and location of germline pathogenic or likely pathogenic variants may influence breast cancer phenotype and survival outcomes. Recent international cohort data suggest that protein-truncating, missense, copy-number, and exon-specific variant patterns may be associated with differences in overall survival and disease-free survival among *BRCA1* and *BRCA2* variant carriers [11]. In parallel, updated gene-specific ACMG/AMP recommendations from the ClinGen ENIGMA *BRCA1* and *BRCA2* Variant Curation Expert Panel provide refined criteria for classifying *BRCA1* and *BRCA2* variants, particularly for resolving variants of uncertain significance [12]. These developments highlight the importance of interpreting *BRCA*-associated prognosis not only according to the affected gene, but also according to variant characteristics and the classification framework used. Our cohort was not powered for variant-level subgroup analysis by variant type or exon location; therefore, available variant-level data for the pathogenic or likely pathogenic *BRCA1* and *BRCA2* variants identified in this cohort are provided in Supplementary Table S1 to facilitate transparent reporting and future pooled or meta-analytic evaluations.

Previous studies evaluating the prognostic role of germline *BRCA* variants in non-metastatic BC have reported inconsistent findings. Retrospective analyses, prospective cohort studies, and meta-analyses have described better, worse, or similar survival outcomes in *BRCA* variant carriers compared with patients with sporadic BC [13,14,15,16,17,18,19]. These discrepancies may partly reflect the frequent grouping of *BRCA1* and *BRCA2* variants into a single category, as well as insufficient adjustment for molecular subtype distribution. A comprehensive meta-analysis concluded that definitive evidence-based conclusions regarding the prognostic impact of *BRCA1* or *BRCA2* carrier status remain limited [17]. Subtype-specific analyses further suggest that the prognostic effect of *BRCA* variants may differ across triple-negative, hormone receptor-positive, and HER2-positive disease [10,20,21]. Therefore, it remains unclear whether the prognostic effects of *BRCA1* and *BRCA2* variants are independent of subtype distribution or reflect intrinsic biological differences between these hereditary tumor groups. Accordingly, this study aimed to evaluate whether germline *BRCA1* and *BRCA2* variants have distinct prognostic associations with disease-free survival and overall survival in patients with non-metastatic stage I–III BC after accounting for molecular subtype and clinicopathological factors.

## 2. Materials and Methods

### 2.1. Study Design and Patient Population

This study was designed as a single-center, retrospective cohort study. Patients were identified from the institutional database of individuals who underwent germline genetic testing at Trakya University Hospital. Among 621 patients tested, those with histologically confirmed invasive breast cancer whose diagnosis, treatment, and follow-up were performed at our institution were screened for eligibility.

Patients diagnosed between 2000 and 2024 were included if they had stage I–III non-metastatic invasive breast cancer at initial diagnosis. Patients with distant metastatic disease at presentation, insufficient clinicopathological or follow-up data, or pathogenic or likely pathogenic germline variants in cancer-predisposition genes other than *BRCA1* or *BRCA2* among the genes included in the testing panel were excluded to reduce potential confounding. According to these criteria, 360 patients were included in the final analysis.

### 2.2. Germline Genetic Analysis

**DNA extraction:** Genomic DNA was extracted from peripheral venous blood samples using the QIAamp DNA Blood Mini Kit (Qiagen, Hilden, Germany), following the manufacturer’s protocol. Germline genetic testing was performed using next-generation sequencing (NGS) and multiplex ligation-dependent probe amplification (MLPA).

**Targeted NGS panels and sequencing:** Two targeted NGS panels were used according to the clinical indication and family history. The *BRCA1* and *BRCA2* Plus Panel (QIAGEN, catalog no. DHS-103Z) covered six genes (*BRCA1*, *BRCA2*, *CDH1*, *PALB2*, *PTEN*, and *TP53*), whereas the QIAseq Targeted DNA Human Breast Cancer Panel (QIAGEN, catalog no. DHS-001Z) covered 93 genes, including *CHEK2*. The complete gene list of the 93-gene panel is provided in the Supplementary Materials.

Supplementary Table S1 presents the detected pathogenic and likely pathogenic *BRCA1* and *BRCA2* variants with available variant-level data, including gene, transcript, nucleotide and protein changes, exon or genomic location, variant type, classification, database identifiers, and detection method.

**Bioinformatic analysis and variant calling**: Read alignment and variant calling were performed using QIAGEN Clinical Insight (QCI) Interpret (QIAGEN, Hilden, Germany; commercial software; https://digitalinsights.qiagen.com/). Candidate variants were visually reviewed in Integrative Genomics Viewer (IGV; Broad Institute; free/open-source software; https://igv.org) to exclude alignment artifacts [22]. Single-nucleotide variants and small insertions/deletions meeting the laboratory’s validated quality and reporting thresholds were reported.

**In silico prediction and variant classification:** Variants were annotated using Franklin (Genoox; https://franklin.genoox.com) and VarSome (https://varsome.com) [23]. Variants were classified according to the 2015 American College of Medical Genetics and Genomics/Association for Molecular Pathology (ACMG/AMP) framework [24] as pathogenic, likely pathogenic, variants of uncertain significance (VUS), likely benign, or benign. Only pathogenic and likely pathogenic *BRCA1* or *BRCA2* variants were used to define carrier status; VUS, likely benign, and benign findings were not considered positive results and were not included in the prognostic carrier groups.

The more recent *BRCA1/BRCA2*-specific ACMG/AMP recommendations issued by the ClinGen ENIGMA Variant Curation Expert Panel [12] were not applied retrospectively. Although pathogenic and likely pathogenic classifications are unlikely to change substantially, some VUS classifications may be revised under the newer gene-specific criteria.

**Databases used for variant interpretation:** Variant interpretation and genotype–phenotype assessment were supported by dbSNP (https://www.ncbi.nlm.nih.gov/snp/; accessed on 16 September 2026) [25], ClinVar (https://www.ncbi.nlm.nih.gov/clinvar/; accessed on 16 September 2026) [26], ClinGen (https://clinicalgenome.org/; accessed on 16 September 2026) [27], the Human Gene Mutation Database (HGMD; https://www.hgmd.cf.ac.uk/; accessed on 16 September 2026 ) [28], and Online Mendelian Inheritance in Man (OMIM; https://www.omim.org/; accessed on 16 September 2026 ) [29].

**MLPA analysis**: In patients without a pathogenic or likely pathogenic single-nucleotide variant or small insertion/deletion detected by NGS, large exonic or whole-gene deletions and duplications in *BRCA1* and *BRCA2* were assessed by multiplex ligation-dependent probe amplification (MLPA) using the SALSA MLPA probemixes P002-D1 (*BRCA1*) and P045-D1 (*BRCA2*) (MRC-Holland, Amsterdam, The Netherlands), according to the manufacturer’s protocol [30]. Fragment data were analyzed using Coffalyser.Net (MRC-Holland; free software; https://www.mlpa.com).

**Gene panel and exclusion criteria**: Patients with a pathogenic or likely pathogenic germline variant in a tested cancer-predisposition gene other than *BRCA1* or *BRCA2* were excluded from the analysis to reduce potential confounding by other hereditary breast cancer syndromes. This exclusion criterion applied only to genes included in the panel used for the individual patient; genes not tested were not considered in the exclusion process.

### 2.3. Collection of Clinicopathological Data

Demographic, clinical, pathological, genetic, treatment, and outcome data were retrospectively extracted from electronic medical records, pathology reports, genetic testing reports, surgical records, and oncology follow-up notes. Recorded variables included age at diagnosis, menopausal status, family history of cancer, tumor laterality and location, tumor size, histological type and grade, clinical or pathological stage, axillary lymph node status, lymphovascular invasion, perineural invasion, skin involvement, surgical margin status, surgical procedure, axillary surgery, systemic therapy, radiotherapy, recurrence, and survival status. The clinicopathological characteristics of the study cohort are presented in Table 1.

Family history of cancer was defined as any documented history of malignant disease in a first- or second-degree relative, including but not limited to breast, ovarian, pancreatic, prostate, and other cancers. This variable reflected the recorded family history of cancer rather than confirmed familial segregation of a cancer-predisposition variant and was therefore interpreted as a clinical background characteristic rather than as proof of inherited pathogenic variant transmission. Data on contralateral risk-reducing mastectomy and risk-reducing salpingo-oophorectomy were not uniformly available and were therefore not included as covariates in the survival models.

### 2.4. Follow-Up

Patients were followed through scheduled oncology outpatient visits and review of electronic medical records. Recurrence data were obtained from clinical notes, imaging reports, pathology reports, and multidisciplinary treatment records. Survival status was determined from hospital records and, when available, telephone follow-up. The last documented clinical contact was used as the censoring date for patients without an event. DFS follow-up time was calculated from the date of curative surgery to the date of recurrence, death, or last follow-up, whereas OS follow-up time was calculated from the date of breast cancer diagnosis to death from any cause or last follow-up. Median follow-up duration was estimated using the reverse Kaplan–Meier method.

### 2.5. Pathological Evaluation and Immunohistochemistry

The diagnosis of invasive breast cancer was confirmed by histopathological examination in all patients. Estrogen receptor (ER) and progesterone receptor (PR) positivity were defined as nuclear staining in ≥1% of tumor cells on immunohistochemistry. HER2 status was assessed according to contemporary ASCO/CAP recommendations used during the relevant diagnostic period. Tumors with 3+ immunohistochemical staining were classified as HER2-positive, whereas equivocal 2+ cases were evaluated by fluorescence in situ hybridization (FISH), and cases with HER2 gene amplification were classified as HER2-positive. The Ki-67 proliferation index was obtained from pathology reports. Although St. Gallen recommendations have used different Ki-67 thresholds over time, including approximately 14% in earlier surrogate subtype definitions and higher thresholds in later consensus discussions [9,31], a 15% cutoff was used for the present retrospective analysis based on the cutoff applied in routine institutional pathology reporting. Given the long study period and the evolution of Ki-67 recommendations over time, this cutoff does not exactly correspond to all St. Gallen consensus thresholds and is acknowledged as a methodological limitation.

### 2.6. Molecular Subtype Classification

Molecular subtypes were assigned using ER, PR, HER2, and Ki-67 results in accordance with St. Gallen surrogate subtype principles [31] and the institutional pathology reporting framework. The following five subgroups were used:

Luminal A: ER positive and/or high PR, HER2 negative, Ki-67 < 15%.

Luminal B/HER2-negative: ER positive, HER2 negative and Ki-67 ≥ 15% or low PR (<20%) or PR negative.

Luminal B/HER2-positive: ER positive and HER2 positive, independent of PR and Ki-67 status.

HER2-positive (non-luminal): ER negative, PR negative and HER2 positive.

Triple-negative: ER negative, PR negative and HER2 negative.

### 2.7. Survival Definitions

The primary endpoints of the study were disease-free survival (DFS) and overall survival (OS).

DFS was defined as the time from the date of curative surgery to the date of first local, regional or distant recurrence or death without recurrence. OS was calculated as the time elapsed from the date of BC diagnosis to death from any cause or the date of the last follow-up. Patients who did not experience an event were censored at the date of the last follow-up.

### 2.8. Statistical Analysis

Continuous variables were summarized as median and minimum–maximum values, and categorical variables were summarized as number and percentage. Group comparisons for categorical variables were performed using the chi-square test or Fisher’s exact test, as appropriate. DFS and OS were estimated using the Kaplan–Meier method, and survival curves were compared with the log-rank test. Pairwise and subgroup comparisons were considered exploratory. Because multiple subgroup and pairwise comparisons were performed, unadjusted *p* values were interpreted cautiously, and statistically significant findings were considered hypothesis-generating unless supported by consistent clinical and multivariable evidence.

Univariate and multivariable Cox proportional hazards regression models were used to identify factors associated with DFS and OS. Variables were not selected solely on the basis of univariate *p* values. Instead, a purposeful selection approach was used: variables with clinical relevance, established prognostic importance, or potential confounding by tumor biology were considered together with univariate results. To reduce collinearity, ER, PR, HER2 status, and histological grade were not entered simultaneously with molecular subtype, because molecular subtype incorporates these biological features. Stage was also not included together with tumor size and nodal status because of conceptual and statistical overlap. Germline *BRCA* variant status was modeled as *BRCA*-negative, *BRCA1*-positive, and *BRCA2*-positive. Treatment-related variables were summarized descriptively but were not included in the primary multivariable models because treatment allocation was closely linked to disease stage, molecular subtype, receptor status, and clinical indication. Because only 27 deaths occurred in the cohort, including 19 deaths in the *BRCA*-negative reference group, the number of OS events was limited relative to the number of covariates included in the multivariable model. Regression methods generally require approximately 5–10 events per variable to avoid biased estimation; therefore, the OS Cox regression results should be interpreted cautiously because of sparse-event bias, model instability, and possible overfitting. The proportional hazards assumption was assessed using graphical inspection of Kaplan–Meier curves and time-dependent patterns; where curves crossed or late risk sets became very small, Cox estimates were interpreted cautiously rather than as definitive evidence. All analyses were performed using IBM SPSS Statistics for Windows, Version 20.0 (IBM Corp., Armonk, NY, USA), and two-sided *p* values < 0.05 were considered statistically significant.

Artificial intelligence (AI) tools were used solely for English-language editing, grammatical refinement, and improvement of the clarity of the manuscript text. AI tools were not used for data analysis, statistical analysis, generation of study results, or scientific decision-making.

### 2.9. Ethics Approval and Consent to Participate

This study was conducted in accordance with the Declaration of Helsinki and approved by the Ethics Committee for Non-Interventional Scientific Research of Trakya University Faculty of Medicine (Approval No: TUTF-GOBAEK 2025/171). Due to the retrospective nature of the study, the requirement for informed consent was waived by the ethics committee.

## 3. Results

### 3.1. Patient Characteristics

A total of 360 patients with non-metastatic stage I–III invasive breast cancer were included in the final analysis. Overall, 281 patients (78.1%) were *BRCA*-negative and 79 patients (21.9%) carried a germline *BRCA* variant, including 43 *BRCA1*-positive and 36 *BRCA2*-positive patients. The median age at diagnosis was 45 years (range, 22–82 years), and the mean age was 46.70 ± 11.53 years. Patients younger than 45 years were more frequent among germline *BRCA* variant carriers than among *BRCA*-negative patients (*p* = 0.008). The median follow-up calculated using the reverse Kaplan–Meier method was 56.26 months (range, 7.30–285.67 months). During follow-up, 76 DFS events were recorded, including 49 events in the *BRCA*-negative group, 11 in the *BRCA1*-positive group, and 16 in the *BRCA2*-positive group. A total of 27 deaths occurred, including 19 in the *BRCA*-negative group, 3 in the *BRCA1*-positive group, and 5 in the *BRCA2*-positive group. Baseline clinicopathological characteristics according to germline *BRCA* variant status are shown in Table 1. A family history of cancer, mastectomy, and stage II–III disease were more frequent among germline *BRCA* variant carriers than among *BRCA*-negative patients.

Several tumor-related characteristics differed according to germline *BRCA* variant status. Grade III tumors and a high Ki-67 proliferation index were more frequent in germline *BRCA* variant carriers than in *BRCA*-negative patients. ER and PR negativity were also more common in the *BRCA*-positive group, particularly among *BRCA1*-positive patients. Molecular subtype distribution differed significantly across *BRCA* subgroups: triple-negative tumors predominated among *BRCA1* variant carriers, whereas hormone receptor-positive luminal subtypes, particularly luminal B disease, were more common among *BRCA2* variant carriers. These descriptive findings support the presence of distinct clinicopathological profiles for *BRCA1*- and *BRCA2*-associated tumors (Figure 1).

### 3.2. Treatment Characteristics

Treatment characteristics are summarized in Table 2 according to germline *BRCA* variant status. Chemotherapy-containing approaches were more frequently used among germline *BRCA* variant carriers, whereas endocrine therapy alone was more common among *BRCA*-negative patients (*p* = 0.002). These treatment patterns are presented descriptively to characterize the cohort, not to infer causal treatment effects on survival. Because treatment selection was strongly influenced by molecular subtype, stage, receptor status, calendar period, and clinical indication, systemic therapy variables were not included in the primary multivariable survival models. The higher frequency of chemotherapy-only treatment among *BRCA1* variant carriers is consistent with the enrichment of triple-negative tumors, whereas the higher frequency of combined chemotherapy and endocrine therapy among *BRCA2* variant carriers is consistent with the predominance of hormone receptor-positive luminal disease.

### 3.3. Disease-Free Survival Analysis

Prognostic factors associated with DFS were evaluated using univariate and multivariable Cox regression analyses (Table 3). In the univariate analysis, tumor size, axillary lymph node status, histological grade, Ki-67 proliferation index, molecular subtype, skin involvement, and *BRCA2* variant status were found to be significantly associated with DFS. These unadjusted associations should be interpreted in the context of the multivariable analysis.

In the multivariable analysis, axillary lymph node status, molecular subtype, and being a *BRCA2* variant carrier were identified as independent prognostic factors for DFS. In particular, the presence of N2 and N3 nodal disease was strongly associated with shorter DFS. In the molecular subtype analysis, luminal B/HER2-negative, HER2-enriched, and triple-negative subtypes were shown to be associated with worse DFS compared to the luminal A subtype. The skin involvement variable, which showed a significant association with DFS in univariate analysis, was not included in the multivariable model due to its strong correlation with tumor stage and potential for collinearity. When germline *BRCA* subtypes were evaluated, Kaplan–Meier analyses showed that DFS was significantly shorter in *BRCA2* variant carriers (Figure 2). Median DFS durations were calculated as 219 months, 225 months, and 132 months in *BRCA*-negative, *BRCA1*-positive, and *BRCA2*-positive patients, respectively. In pairwise comparisons using the log-rank test, it was found that DFS was significantly shorter in *BRCA2*-positive patients compared to *BRCA*-negative patients (*p* = 0.015), while no significant difference was observed between the *BRCA1*-positive and *BRCA*-negative groups (*p* = 0.788). In multivariable analysis, carrying a *BRCA2* variant was identified as an independent negative prognostic factor for DFS, while no independent association was found between *BRCA1* variant status and DFS. Variables included in the multivariable analyses, such as tumor size, Ki-67 proliferation index, and perineural invasion, were not among the independent prognostic factors for DFS.

### 3.4. Overall Survival Analysis

OS analyses are presented in Table 4. In univariate analysis, tumor size, axillary lymph node status, skin involvement, perineural invasion, surgical margin status, chemotherapy timing, and molecular subtype were found to be significantly associated with OS. Treatment-related variables such as chemotherapy timing were not included in multivariable analyses as they are considered to be factors primarily reflecting disease burden and clinical treatment indications and do not directly represent intrinsic tumor biology.

In multivariable analysis, tumor size, axillary lymph node status, and skin involvement were identified as independent prognostic factors for OS. Tumor size > 5 cm was associated with shorter OS, and N2 disease was independently associated with OS. Skin involvement was also independently associated with shorter OS. For molecular subtype, luminal B/HER2-negative and triple-negative subtypes showed worse OS, although these associations were borderline in the multivariable analysis. Crude Kaplan–Meier analyses showed no significant difference in OS according to germline *BRCA* variant status (Figure 3); the log-rank and pairwise findings should be regarded as exploratory. In the multivariable Cox model, *BRCA1* variant status was associated with a lower estimated hazard of death (HR 0.08, 95% CI 0.01–0.51, *p* = 0.007). Given the limited number of deaths, this estimate should be interpreted cautiously and should not be considered definitive evidence of improved survival. *BRCA2* variant status was not independently associated with OS. During follow-up, unadjusted comparisons showed that recurrence and distant metastasis were more frequent among patients with a germline BRCA variant than among BRCA-negative patients (Table 2); these observational comparisons do not establish a gene-specific effect and were further examined in the DFS and OS models (Table 3 and Table 4).

## 4. Discussion

In this study, we showed that the prognostic effects of germline *BRCA1* and *BRCA2* variant in patients with non-metastatic invasive BC are not homogeneous, and that *BRCA1* and *BRCA2* show different survival patterns when molecular subtypes and established clinicopathological factors are considered. Our most striking finding is that while germline *BRCA2* variant carrier status was independently associated with worse DFS, *BRCA1* variant carrier status was associated with a lower estimated hazard of death in the multivariable OS model; however, this finding should be interpreted cautiously given the limited number of death events. These results suggest that considering *BRCA1* and *BRCA2* variants as a single biological group may be misleading when making prognostic assessments in non-metastatic BC.

The prognostic role of *BRCA* variants in non-metastatic BC has been controversial in the current literature, with conflicting results reported in different studies [3,4,13,32,33]. Even in comprehensive systematic reviews and meta-analyses, no clear conclusion has been reached regarding the effect of being a *BRCA* variant carrier on survival, highlighting the importance of this heterogeneity [17,18,34]. Several factors may contribute to this inconsistency, including the frequent evaluation of *BRCA1* and *BRCA2* variants as a single group, insufficient consideration of molecular subtype, and treatment-related confounding.

In our study, the separate evaluation of *BRCA1* and *BRCA2* variants using multivariable analyses adjusted for molecular subtype and clinicopathological variables helps to clarify this uncertainty in the literature. While the predominance of the triple-negative subtype in *BRCA1* variant carriers and the more frequent observation of luminal subtypes in *BRCA2* variant carriers are consistent with the literature, the fact that *BRCA2* variant carrier status remained independently associated with worse DFS after inclusion of molecular subtype in the multivariable model suggests that this association may not be explained solely by differences in molecular subtype distribution [35,36,37].

*BRCA2* variants are known to be more frequently associated with hormone receptor-positive and HER2-negative BC [5,7,8]. However, increasing evidence suggests that *BRCA2* variant-associated luminal tumors may exhibit clinical and biological features that differ from those of sporadic luminal tumors. Genomic instability and proliferative activity have been proposed as potential biological mechanisms that may contribute to differences in clinical behavior. In our study, *BRCA2* variant carrier status was independently associated with shorter DFS after adjustment for molecular subtype and other clinicopathological factors. Some retrospective cohort studies in the literature have also reported an increased risk of early relapse and shorter DFS, especially in hormone receptor-positive *BRCA2* variant carriers [38,39]. Taken together, these observations suggest that molecular subtype alone may not fully account for the association between *BRCA2* variant status and DFS observed in our cohort.

Another important finding of our study was that *BRCA1* variant status was associated with a lower estimated hazard of death in the multivariable OS model. However, this finding should be interpreted cautiously given the limited number of death events and the resulting risk of model instability and overfitting. The prognostic effect of *BRCA* variants in non-metastatic BC is still controversial in the literature, with conflicting results reported in different studies. While some studies have reported worse survival in patients carrying *BRCA1* variants [18,19,34], other studies have not shown a significant survival difference between variant carriers and non-carriers [13,40]. Therefore, the observed association between *BRCA1* variant status and lower mortality hazard in our cohort should be considered exploratory rather than definitive evidence of a survival advantage and requires validation in larger cohorts with more OS events.

Emerging evidence suggests that prognostic heterogeneity among *BRCA1* and *BRCA2* carriers may also be influenced by variant type and intragenic location. A recent international cohort study reported differences in breast cancer survival according to protein-truncating versus missense variants and variant location, suggesting that variant-level characteristics may further modify prognosis among *BRCA1* and *BRCA2* carriers [11].

Molecular subtypes are among the strongest predictors of survival in non-metastatic BC [10,39]. In our study, luminal B/HER2-negative, HER2-enriched, and triple-negative subtypes were shown to be associated with a worse prognosis in terms of DFS compared to the luminal A subtype. However, the prognostic effects of *BRCA* variants persisted after adjustment for molecular subtype and other clinicopathological factors. This suggests that the gene-specific associations observed for *BRCA1* and *BRCA2* may not be fully explained by differences in molecular subtype distribution alone. The differences in treatment patterns observed across germline *BRCA* groups should also be interpreted in the context of the distinct distribution of molecular subtypes. As systemic treatment selection is strongly influenced by hormone receptor and HER2 status, the different molecular subtype distributions among *BRCA1*, *BRCA2*, and BRCA-negative patients may have contributed to the observed differences in treatment characteristics and represent a potential source of confounding. Accordingly, treatment comparisons in the present study should be regarded as descriptive rather than causal.

The strengths of this study include the separate evaluation of *BRCA1* and *BRCA2* variants in a homogeneous non-metastatic breast cancer patient population and the inclusion of molecular subtype and relevant clinicopathological factors in the survival analyses. However, the single-center and retrospective design, the inclusion of only patients who underwent germline testing, with the potential for selection and referral bias, the limited number of patients in subgroup analyses, and the relatively low number of OS events are the main limitations of the study. In particular, only 27 deaths occurred in the entire cohort, resulting in a limited number of events relative to the number of covariates in the OS model and increasing the risk of model instability and possible overfitting. Therefore, the OS findings, particularly the association observed for *BRCA1* variant status, should be considered exploratory. In addition, systematic adjustment for multiple comparisons was not performed; therefore, subgroup and pairwise comparisons should be considered exploratory and interpreted cautiously. Data on platinum-specific chemotherapy exposure were not available. In addition, data on risk-reducing procedures, including contralateral risk-reducing mastectomy (CRRM) and risk-reducing salpingo-oophorectomy (RRSO), as well as the timing of these procedures, were not systematically collected. Consequently, these factors could not be incorporated into the survival models, and residual confounding related to systemic treatment and risk-reducing surgical interventions cannot be excluded. Furthermore, the absence of PARP inhibitor use in the adjuvant phase may limit the generalizability of these results to current treatment regimens. The long study period from 2000 to 2024, which encompassed changes in diagnostic approaches, germline testing practices, and treatment standards, represents an additional potential source of heterogeneity. These findings need to be validated in larger, prospective cohorts. Overall, our findings support the separate evaluation of *BRCA1* and *BRCA2* variants in prognostic research. In particular, the association between *BRCA2* variant status and worse DFS warrants further investigation, whereas the apparent association between *BRCA1* variant status and lower mortality hazard requires cautious interpretation and validation in cohorts with a larger number of OS events.

## 5. Conclusions

In this retrospective real-world cohort of patients with non-metastatic stage I–III breast cancer, germline *BRCA1* and *BRCA2* variants showed distinct prognostic associations rather than representing a single homogeneous prognostic category. *BRCA2* variant status was independently associated with worse DFS, whereas *BRCA1* variant status was associated with a lower estimated hazard of death in the multivariable OS model. However, the OS finding should be considered exploratory and interpreted cautiously because of the limited number of death events and the potential for model instability and overfitting. These findings support the separate evaluation of *BRCA1* and *BRCA2* variant status in prognostic research. Larger multicenter prospective studies with adequate OS events, standardized follow-up, and detailed treatment and variant-level data are needed to validate these associations before they can inform individual risk stratification or clinical management.

## Figures and Tables

**Figure 1:**
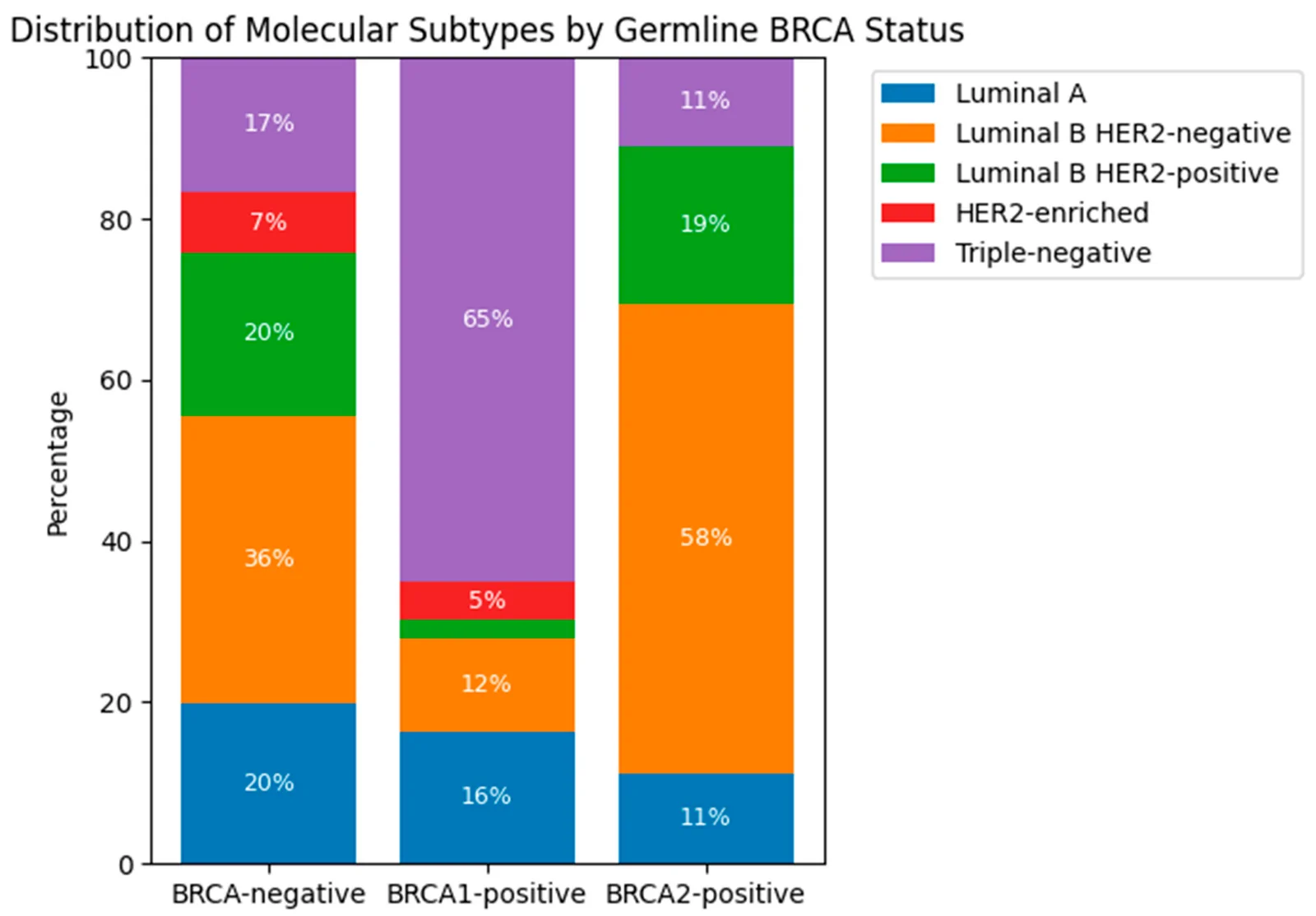
**Distribution of molecular subtypes according to germline BRCA variant status.** Figure legend: The distribution of molecular subtypes differed significantly across BRCA subgroups. Triple-negative tumors were markedly more frequent among *BRCA1* variant carriers, whereas luminal subtypes (particularly luminal B) predominated among *BRCA2* variant carriers. These findings highlight the distinct biological characteristics associated with *BRCA1* and *BRCA2* variants.

**Figure 2:**
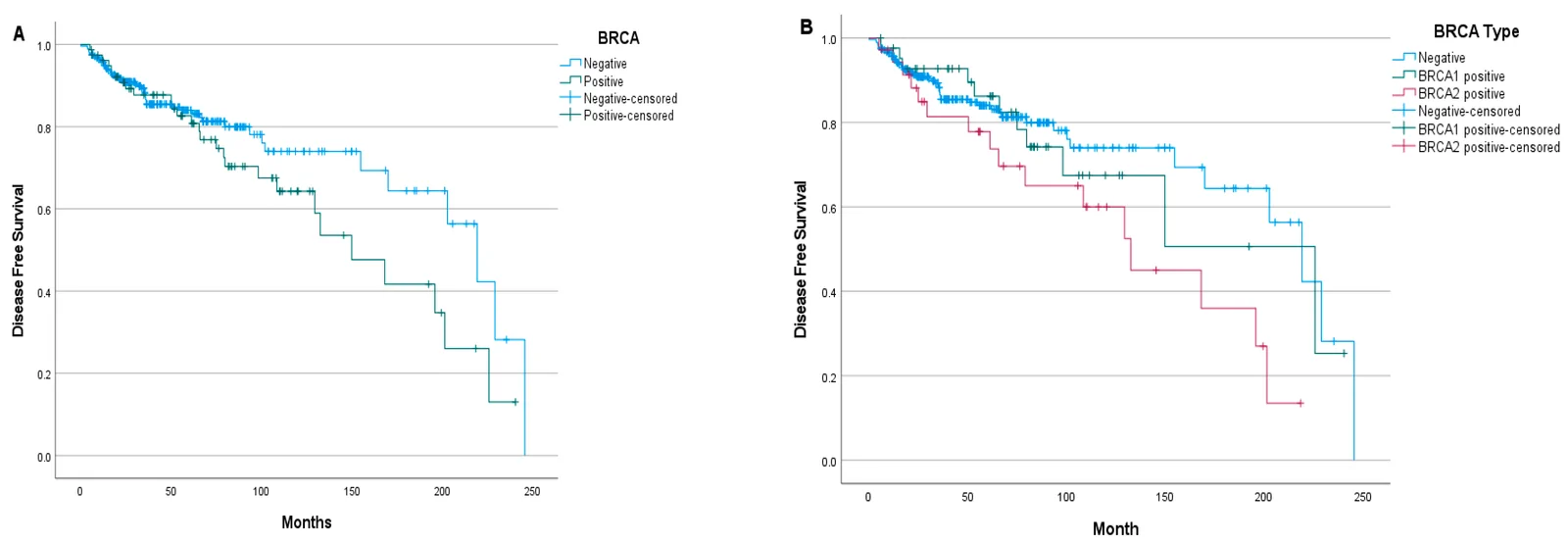
**Kaplan–Meier curves for disease-free survival according to germline BRCA variant status.** (**A**) Disease-free survival according to BRCA variant status (negative vs. positive). (**B**) Disease-free survival according to BRCA subtype (*BRCA*-negative, *BRCA1*-positive, and *BRCA2*-positive). Log-rank *p* = 0.099 (**A**) and *p* = 0.059 (**B**).

**Figure 3:**
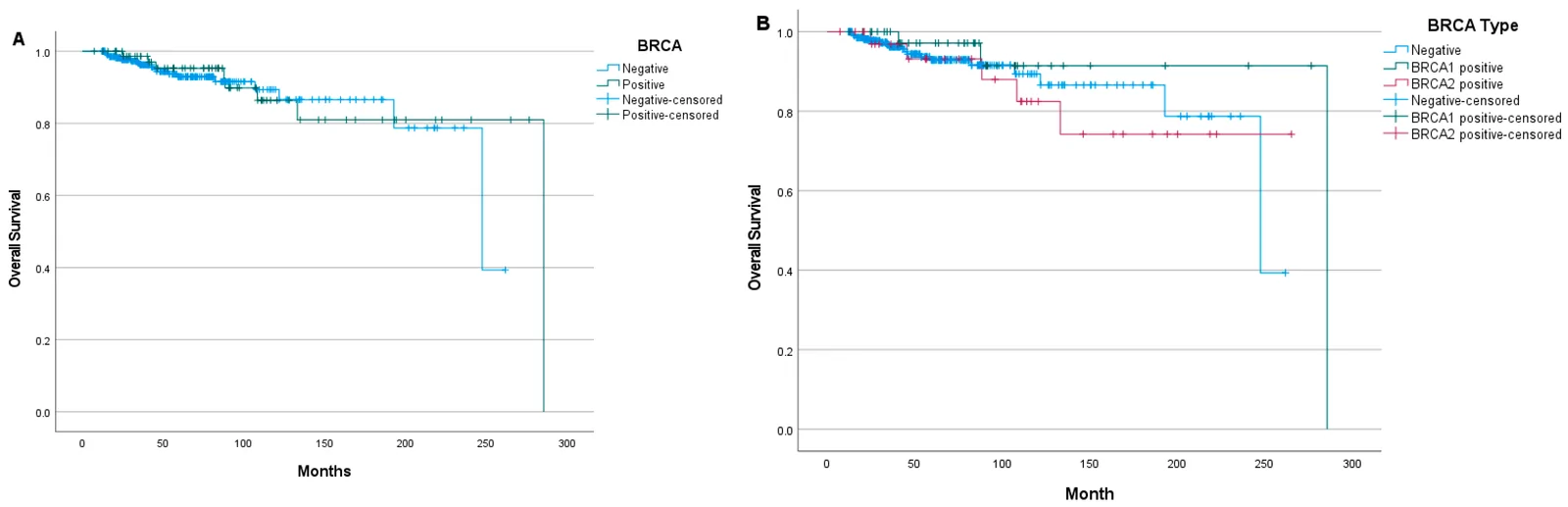
**Kaplan–Meier curves for overall survival according to germline BRCA variant status.** (**A**) Overall survival according to *BRCA* variant status (*BRCA*-negative, *n* = 281; *BRCA*-positive, *n* = 79). (**B**) Overall survival according to *BRCA* subtype (*BRCA*-negative, *n* = 281; *BRCA1*-positive, *n* = 43; and *BRCA2*-positive, *n* = 36). Log-rank *p* = 0.687 (**A**) and *p* = 0.468 (**B**). The color legend in each panel identifies the corresponding patient groups. *p*-values are from the log-rank test, and pairwise comparisons are considered exploratory. The late decline to 0% survival reflects the death of the final patient remaining at risk at 285.67 months; therefore, the extreme tail of the curve should be interpreted cautiously.

**Table 1:** Clinicopathological characteristics of non-metastatic breast cancer patients stratified by germline BRCA variant status and BRCA subtype.

		*BRCA*					BRCA Status				
		Negative		Positive			*BRCA1*-Positive		*BRCA2*-Positive		
		*n*	%	*n*	%	*p*	*n*	%	*n*	%	*p*
Age at diagnosis	<45 years	127	45.2	49	62.0	0.008	28	65.1	21	58.3	0.700
	≥45 years	154	54.8	30	38.0		15	34.9	15	41.7	
Patient sex	Female	276	98.2	79	100.0	0.590	43	100.0	36	100.0	–
	Male	5	1.8	0	0.0		0	0.0	0	0.0	
Family history of cancer	No	194	69.0	29	36.7	<0.001	16	37.2	13	36.1	1.000
	Yes	87	31.0	50	63.3		27	62.8	23	63.9	
Menopausal status	Premenopausal	159	57.6	55	69.6	0.054	30	69.8	25	69.4	1.000
	Postmenopausal	117	42.4	24	30.4		13	30.2	11	30.6	
Surgery type	Breast-conserving surgery (BCS)	184	65.5	33	41.8	<0.001	18	41.9	15	41.7	1.000
	Mastectomy	97	34.5	46	58.2		25	58.1	21	58.3	
Axillary surgery	Sentinel lymph node biopsy (SLNB)	145	51.6	29	36.7	0.019	14	32.6	15	41.7	0.547
	Axillary lymph node dissection (ALND)	136	48.4	50	63.3		29	67.4	21	58.3	
Anatomical location	Left breast	139	49.5	37	46.8	0.677	21	48.8	16	44.4	0.731
	Right breast	136	48.4	39	49.4		21	48.8	18	50.0	
	Bilateral	6	2.1	3	3.8		1	2.3	2	5.6	
Tumor quadrant	Inner quadrant	46	16.4	16	20.3	0.169	9	20.9	7	19.4	0.352
	Outer quadrant	179	63.7	54	68.4		30	69.8	24	66.7	
	Central	40	14.2	4	5.1		3	7.0	1	2.8	
	Multicentric	16	5.7	5	6.3		1	2.3	4	11.1	
Laterality	Unilateral	269	95.7	75	94.9	0.759	42	97.7	33	91.7	0.326
	Bilateral	12	4.3	4	5.1		1	2.3	3	8.3	
Tumor size (cm)	≤2	112	39.9	19	24.1	0.032	10	23.3	9	25.0	0.561
	>2–5	145	51.6	50	63.3		26	60.5	24	66.7	
	>5	24	8.5	10	12.7		7	16.3	3	8.3	
Axillary nodal status	N0	132	47.0	35	44.3	0.969	20	46.5	15	41.7	0.541
	N1	84	29.9	24	30.4		12	27.9	12	33.3	
	N2	53	18.9	16	20.3		10	23.3	6	16.7	
	N3	12	4.3	4	5.1		1	2.3	3	8.3	
Clinical or pathological stage	Stage I	79	28.1	9	11.4	0.007	5	11.6	4	11.1	0.563
	Stage II	128	45.6	48	60.8		24	55.8	24	66.7	
	Stage III	74	26.3	22	27.8		14	32.6	8	22.2	
Histological type of tumor	IDC	248	88.3	70	88.6	0.339	39	90.7	31	86.1	0.139
	ILC	11	3.9	2	2.5		1	2.3	1	2.8	
	IDC + ILC	3	1.1	3	3.8		0	0.0	3	8.3	
	Other	19	6.8	4	5.1		3	7.0	1	2.8	
Tumor grade	Grade I–II	173	61.6	30	38.0	<0.001	10	23.3	20	55.6	0.007
	Grade III	108	38.4	49	62.0		33	76.7	16	44.4	
Ki-67 index	<15%	99	35.2	15	19.0	0.006	8	18.6	7	19.4	1.000
	≥15%	182	64.8	64	81.0		35	81.4	29	80.6	
ER status	Negative	74	26.3	34	43.0	0.004	30	69.8	4	11.1	<0.001
	Positive	207	73.7	45	57.0		13	30.2	32	88.9	
PR status	Negative	110	39.1	42	53.2	0.026	32	74.4	10	27.8	<0.001
	Positive	171	60.9	37	46.8		11	25.6	26	72.2	
HER2 status	Negative	204	72.6	69	87.3	0.007	40	93.0	29	80.6	0.172
	Positive	77	27.4	10	12.7		3	7.0	7	19.4	
Molecular subtype	Luminal A	56	19.9	11	13.9	<0.001	7	16.3	4	11.1	<0.001
	Luminal B HER2-negative	100	35.6	26	32.9		5	11.6	21	58.3	
	Luminal B HER2-positive	57	20.3	8	10.1		1	2.3	7	19.4	
	HER2-enriched	21	7.5	2	2.5		2	4.7	0	0.0	
	Triple-negative	47	16.7	32	40.5		28	65.1	4	11.1	
Lymphovascular invasion	Absent	163	58.0	45	57.0	0.868	23	53.5	22	61.1	0.650
	Present	118	42.0	34	43.0		20	46.5	14	38.9	
Perineural invasion	Absent	233	82.9	67	84.8	0.690	37	86.0	30	83.3	0.738
	Present	48	17.1	12	15.2		6	14.0	6	16.7	
Skin involvement	Absent	268	95.4	76	96.2	1.000	40	93.0	36	100.0	0.246
	Present	13	4.6	3	3.8		3	7.0	0	0.0	
Surgical margin	Negative	249	88.6	69	87.3	0.756	37	86.0	32	88.9	0.748
	Positive	32	11.4	10	12.7		6	14.0	4	11.1	

**Abbreviations**: BRCA, breast cancer susceptibility gene; BCS, breast-conserving surgery; SLNB, sentinel lymph node biopsy; ALND, axillary lymph node dissection; IDC, invasive ductal carcinoma; ILC, invasive lobular carcinoma; ER, estrogen receptor; PR, progesterone receptor; HER2, human epidermal growth factor receptor 2. **Footnotes**: Data are presented as *n* (column %) unless otherwise specified. Percentages were calculated within columns based on available data for each variable unless otherwise specified. *p* values were calculated using the chi-square test or Fisher’s exact test, as appropriate. *p* values for comparisons between *BRCA1* and *BRCA2* subgroups were calculated using Fisher’s exact test; due to small sample sizes in some categories (particularly molecular subtypes), these results should be interpreted with caution. *p* values were not calculated for variables with insufficient cell counts.

**Table 2:** Treatment and outcome characteristics of non-metastatic breast cancer patients stratified by germline BRCA variant status and BRCA subtype.

		BRCA					BRCA_Status				
		Negative		Positive			*BRCA1*-Positive		*BRCA2*-Positive		
		*n*	%	*n*	%	*p*	*n*	%	*n*	%	*p*
Systemic therapy type	Chemotherapy alone	69	24.6	33	41.8	0.002	29	67.4	4	11.1	<0.001
	Endocrine therapy alone	48	17.1	4	5.1		2	4.7	2	5.6	
	Chemotherapy and endocrine therapy	164	58.4	42	53.2		12	27.9	30	83.3	
Chemotherapy use	Not received	48	17.1	4	5.1	0.012	2	4.7	2	5.6	1.000
	Received	233	82.9	75	94.9		41	95.3	34	94.4	
Chemotherapy timing	Neoadjuvant	95	40.8	26	34.7	0.346	15	36.6	11	32.4	0.899
	Adjuvant	138	59.2	49	65.3		26	63.4	23	67.6	
Type of chemotherapy regimen (if chemotherapy received)	Anthracycline + taxane	186	79.8	64	86.5	0.027	37	92.5	27	79.4	0.171
	Anthracycline-based	26	11.2	10	13.5		3	7.5	7	20.6	
	Taxane-based	21	9.0	0	0.0		0	0.0	0	0.0	
Use of trastuzumab	Not received	204	72.6	69	87.3	0.007	40	93.0	29	80.6	0.172
	Received	77	27.4	10	12.7		3	7.0	7	19.4	
Radiotherapy	Not received	14	5.0	6	7.6	0.404	2	4.7	4	11.1	0.403
	Received	267	95.0	73	92.4		41	95.3	32	88.9	
Radiotherapy type	Breast only	77	28.8	10	13.7	0.013	5	12.2	5	15.6	0.740
	Locoregional	190	71.2	63	86.3		36	87.8	27	84.4	
Hormone therapy	Not received	69	24.6	33	41.8	0.003	29	67.4	4	11.1	<0.001
	Received	212	75.4	46	58.2		14	32.6	32	88.9	
Type of hormone therapy agent	Tamoxifen	99	46.7	20	43.5	0.205	7	50.0	13	40.6	0.742
	Letrozole	22	10.4	7	15.2		2	14.3	5	15.6	
	Anastrozole	60	28.3	8	17.4		3	21.4	5	15.6	
	Tamoxifen and AI sequentially	31	14.6	11	23.9		2	14.3	9	28.1	
Tamoxifen duration	<5 years	91	70.5	16	51.6	0.072	3	33.3	13	59.1	0.252
	≥5 years	38	29.5	15	48.4		6	66.7	9	40.9	
AI duration	<5 years	77	71.3	14	58.3	0.319	3	42.9	11	64.7	0.393
	≥5 years	31	28.7	10	41.7		4	57.1	6	35.3	
LHRH therapy duration	<2 years	48	41.0	11	39.3	1.000	3	37.5	8	40.0	1.000
	2–5 years	69	59.0	17	60.7		5	62.5	12	60.0	
Local nodal recurrence	No	269	95.7	74	93.7	0.547	41	95.3	33	91.7	0.655
	Yes	12	4.3	5	6.3		2	4.7	3	8.3	
Presence of distant metastasis	No	237	84.3	55	69.6	0.003	35	81.4	20	55.6	0.013
	Yes	44	15.7	24	30.4		8	18.6	16	44.4	
Recurrence status	No recurrence	232	82.6	52	65.8	0.001	32	74.4	20	55.6	0.078
	Recurrence present	49	17.4	27	34.2		11	25.6	16	44.4	
Vital status	Alive	262	93.2	71	89.9	0.316	40	93.0	31	86.1	0.458
	Died	19	6.8	8	10.1		3	7.0	5	13.9	

**Abbreviations**: BRCA, breast cancer susceptibility gene; AI, aromatase inhibitor; LHRH, luteinizing hormone-releasing hormone. **Footnotes**: Data are presented as *n* (column %) unless otherwise specified. For treatment timing, regimen, type, agent, and duration variables, percentages were calculated using the relevant treated subgroup with available data as the denominator. *p* values were calculated using the chi-square test or Fisher’s exact test, as appropriate. *p* values for comparisons between *BRCA1* and *BRCA2* subgroups were calculated using Fisher’s exact test. *p* values were not calculated for variables with insufficient cell counts.

**Table 3:** Univariate and Multivariable Cox Regression Analysis for Disease–Free Survival (DFS).

Covariate	Univariate Analysis			Multivariable Analysis		
	HR	95% CI	*p*	HR	95% CI	*p*
Age at diagnosis (years)						
<45	1 (reference)	–	–	–	–	–
≥45	0.78	0.50–1.24	0.309			
Tumor size (cm)						
≤2	1 (reference)	–	–	1 (reference)	–	–
>2–5 cm	2.00	1.14–3.50	0.014	1.31	0.71–2.42	0.381
>5 cm	3.93	1.99–7.76	<0.001	2.08	0.95–4.53	0.065
Axillary nodal status						
N0	1 (reference)	–	–	1 (reference)	–	–
N1	1.64	0.88–3.06	0.117	1.67	0.84–3.30	0.140
N2	5.64	3.08–10.30	<0.001	6.27	3.13–12.56	<0.001
N3	5.98	2.58–13.87	<0.001	4.07	1.56–10.63	0.004
Tumor grade						
Grade I–II	1 (reference)	–	–	–	–	–
Grade III	1.77	1.12–2.80	0.014			
Ki-67 index						
<15%	1 (reference)	–	–	1 (reference)	–	–
≥15%	3.02	1.65–5.53	<0.001	1.36	0.64–2.88	0.415
ER status						
Negative	1 (reference)	–	–	–	–	–
Positive	0.65	0.40–1.03	0.068			
PR status						
Negative	1 (reference)	–	–	–	–	–
Positive	0.58	0.36–0.91	0.020			
HER2 status						
Negative	1 (reference)	–	–	–	–	–
Positive	0.77	0.42–1.41	0.404			
Molecular subtype						
Luminal A	1 (reference)	–	–	1 (reference)	–	–
Luminal B HER2−	7.50	2.63–21.40	<0.001	4.57	1.33–15.68	0.015
Luminal B HER2+	3.89	1.17–12.88	0.026	1.99	0.49–7.94	0.330
HER2–enriched	4.69	1.16–18.94	0.030	5.73	1.25–26.24	0.025
Triple–negative	6.77	2.34–19.59	<0.001	5.98	1.63–21.90	0.007
Lymphovascular invasion						
Absent	1 (reference)	–	–	–	–	–
Present	1.24	0.78–1.97	0.348			
Perineural invasion						
Absent	1 (reference)	–	–	1 (reference)	–	–
Present	1.66	0.99–2.80	0.054	1.43	0.82–2.48	0.197
Skin involvement						
Absent	1 (reference)	–	–	–	–	–
Present	5.24	2.48–11.07	<0.001			
Surgical margin						
Negative	1 (reference)	–	–	–	–	–
Positive	1.69	0.94–3.04	0.077			
Radiotherapy type						
Breast only	1 (reference)	–	–	–	–	–
Locoregional	3.49	1.67–7.29	0.001			
BRCA						
Negative	1 (reference)	–	–	–	–	–
Positive	1.48	0.92–2.39	0.101			
BRCA type						
Negative	1 (reference)	–	–	1 (reference)	–	–
*BRCA1*-positive	1.09	0.56–2.12	0.786	0.70	0.32–1.49	0.359
*BRCA2*-positive	1.98	1.11–3.52	0.020	2.13	1.14–3.98	0.017

**Abbreviations**: HR, hazard ratio; CI, confidence interval; DFS, disease-free survival; ER, estrogen receptor; PR, progesterone receptor; HER2, human epidermal growth factor receptor 2; BRCA, breast cancer susceptibility gene. **Footnotes**: Variables were selected for inclusion in the multivariable model based on clinical relevance, established prognostic importance, potential confounding, and univariate findings. Molecular subtype was included to account for tumor biology, and ER, PR, HER2, and histological grade were excluded due to collinearity. Tumor stage was excluded due to its overlap with tumor size and nodal status. Treatment-related variables were not included in the primary multivariable model because of potential confounding by clinical indication and tumor characteristics.

**Table 4:** Univariate and Multivariable Cox Regression Analysis for Overall Survival (OS).

Covariate	Univariate Analysis			Multivariable Analysis		
	HR	95% CI	*p*	HR	95% CI	*p*
Age at diagnosis (years)						
<45	1 (reference)	–	–	–	–	–
≥45	0.74	0.34–1.62	0.462			
Tumor size (cm)						
≤2	1 (reference)	–	–	1 (reference)	–	–
>2–5 cm	3.16	1.01–9.86	0.047	1.98	0.53–7.37	0.306
>5 cm	9.62	2.79–33.08	<0.001	4.70	1.08–20.38	0.038
Axillary nodal status						
N0	1 (reference)	–	–	1 (reference)	–	–
N1	1.35	0.40–4.49	0.619	1.10	0.28–4.32	0.890
N2	7.81	2.78–21.96	<0.001	6.83	1.91–24.39	0.003
N3	4.45	0.86–23.01	0.075	2.02	0.30–13.59	0.469
Tumor grade						
Grade I–II	1 (reference)	–	–	–	–	–
Grade III	1.66	0.75–3.68	0.206			
Ki-67 index						
<15%	1 (reference)	–	–	1 (reference)	–	–
≥15%	2.52	0.92–6.87	0.071	1.15	0.32–4.09	0.828
ER status						
Negative	1 (reference)	–	–	–	–	–
Positive	0.49	0.22–1.06	0.072			
PR status						
Negative	1 (reference)	–	–	–	–	–
Positive	0.47	0.21–1.06	0.070			
HER2 status						
Negative	1 (reference)	–	–	–	–	–
Positive	0.43	0.13–1.46	0.178			
Molecular subtype						
Luminal A	1 (reference)	–	–	1 (reference)	–	–
Luminal B HER2−	11.53	1.43–92.49	0.021	7.65	0.70–83.35	0.095
Luminal B HER2+	3.28	0.27–38.67	0.345	1.41	0.08–22.45	0.806
HER2–enriched	5.32	0.31–89.80	0.246	5.66	0.23–135.99	0.285
Triple–negative	11.87	1.40–100.40	0.023	11.93	0.87–162.46	0.063
Lymphovascular invasion						
Absent	1 (reference)	–	–	–	–	–
Present	1.42	0.64–3.13	0.377			
Perineural invasion						
Absent	1 (reference)	–	–	1 (reference)	–	–
Present	2.71	1.19–6.15	0.017	1.93	0.79–4.70	0.146
Skin involvement						
Absent	1 (reference)	–	–	1 (reference)	–	–
Present	11.94	4.66–30.58	<0.001	9.19	2.83–29.83	<0.001
Surgical margin						
Negative	1 (reference)	–	–	–	–	–
Positive	2.68	1.11–6.47	0.028			
Chemotherapy timing						
Neoadjuvant	1 (reference)	–	–	–	–	–
Adjuvant	0.25	0.10–0.57	0.001			
BRCA type						
Negative	1 (reference)	–	–	1 (reference)	–	–
*BRCA1* positive	0.45	0.10–1.98	0.297	0.08	0.01–0.51	0.007
*BRCA2* positive	1.24	0.45–3.38	0.671	1.60	0.48–5.29	0.441

**Abbreviations:** HR, hazard ratio; CI, confidence interval; OS, overall survival; ER, estrogen receptor; PR, progesterone receptor; HER2, human epidermal growth factor receptor 2; BRCA, breast cancer susceptibility gene. **Footnotes:** Variables with *p* < 0.05 in univariate analysis and clinically relevant variables were included in the multivariable model. Molecular subtype was included to represent tumor biology, while ER, PR, HER2, and histological grade were excluded due to collinearity. Treatment-related variables were not included to avoid treatment-related confounding. Tumor stage was not included due to its strong correlation with tumor size and nodal status. Given the limited number of events, the results of the multivariable analysis should be interpreted with caution.

## Data Availability

The datasets generated and/or analyzed during the current study are available from the corresponding author upon reasonable request.

## Supplementary Materials

The following supporting information can be downloaded at https://www.mdpi.com/article/10.3390/cancers18193073/s1: Table S1: Germline *BRCA1* and *BRCA2* pathogenic and likely pathogenic variants identified in the study cohort.
