# Supplementary material for: Divergent Prognostic Impact of Germline BRCA1 and BRCA2 Variants in Non-Metastatic Breast Cancer: A Real-World Cohort Study
Source: Cancers (Basel). 2026 Sep 22;18(19):3073. doi: 10.3390/cancers18193073 (PMC13664164; doi:10.3390/cancers18193073)
Supplement: Supplementary file 1 [file cancers-18-03073-s001.zip › cancers-4544848-supplementary.pdf]

Supplementary Table S1. Germline *BRCA1* and *BRCA2* Pathogenic and Likely Pathogenic Variants Identified in the Study Cohort

Carrier No.	Gene	Transcript	HGVS c.	HGVS p.	Exon / location	Variant type	ACMG classification	dbSNP / ClinVar ID	Method
C01	BRCA1	NM_007294.4	c.116G>A	NP_009225.1:p.Cys39Tyr	3	Missense	Pathogenic	rs80357498 / 37392	NGS
C02	BRCA1	NM_007294.4	c.1789G>T	NP_009228.2:p.Glu597Ter	10	Nonsense	Pathogenic	rs55650082 / 54348	NGS
C03	BRCA1	NM_007294.4	c.1789G>T	NP_009228.2:p.Glu597Ter	10	Nonsense	Pathogenic	rs55650082 / 54348	NGS
C04	BRCA1	NM_007294.4	c.181T>G	NP_009225.1:p.Cys61Gly	4	Missense	Pathogenic	rs28897672 / 17661	NGS
C05	BRCA1	NM_007294.4	c.1885A>T	NP_009225.1:p.Arg629Ter	10	Nonsense	Pathogenic	rs1555590987 / 548273	NGS
C06	BRCA1	NM_007294.4	c.1885A>T	NP_009225.1:p.Arg629Ter	10	Nonsense	Pathogenic	rs1555590987 / 548273	NGS
C07	BRCA1	NM_007294.4	c.2611_2612delCC	NP_009225.1:p.Pro871ValfsTer31	10	Frameshift	Pathogenic	rs80357962 / 54617	NGS
C08	BRCA1	NM_007294.4	c.2952delT	NP_009225.1:p.Ile986Serfs*14	10	Frameshift	Pathogenic	rs80357627 / 54729	NGS
C09	BRCA1	NM_007294.4	c.302-2A>C	NA	Intron 5	Splicing	Pathogenic	rs80358011 / 37500	NGS
C10	BRCA1	NM_007294.4	c.3607C>T	NP_009225.1:p.Arg1203Ter	10	Nonsense	Pathogenic	rs62625308 / 17671	NGS
C11	BRCA1	NM_007294.4	c.3700_3704delGTAAA	NP_009225.1:p.Val1234Glnfs*8	10	Frameshift	Pathogenic	rs80357609 / 37542	NGS
C12	BRCA1	NM_007294.4	c.3700_3704delGTAAA	NP_009225.1:p.Val1234Glnfs*8	10	Frameshift	Pathogenic	rs80357609 / 37542	NGS
C13	BRCA1	NM_007294.4	c.3700_3704delGTAAA	NP_009225.1:p.Val1234Glnfs*8	10	Frameshift	Pathogenic	rs80357609 / 37542	NGS
C14	BRCA1	NM_007294.4	c.3700_3704delGTAAA	NP_009225.1:p.Val1234Glnfs*8	10	Frameshift	Pathogenic	rs80357609 / 37542	NGS
C15	BRCA1	NM_007294.4	c.3700_3704delGTAAA	NP_009225.1:p.Val1234Glnfs*8	10	Frameshift	Pathogenic	rs80357609 / 37542	NGS
C16	BRCA1	NM_007294.3:	c.4393delA	NP_009225.1:p.Pro1464_Ile1465insTer	13	Frameshift	Pathogenic	rs397507230 / 37590	NGS
C17	BRCA1	NM_007294.4:	c.4566C>A	NP_009225.1: p.Tyr1522Ter	NA	Nonsense	Pathogenic	rs886040234 / 266480	NGS
C18	BRCA1	NM_007294.4	c.4566C>A	NP_009225.1:p.Tyr1522Ter	14	Nonsense	Pathogenic	rs886040234 / 266480	NGS
C19	BRCA1	NM_007294.3	c.4818del	NP_009225.1:p.Val1607Leufs*26	15	Frameshift	Pathogenic	rs1555580883 / 495093	NGS
C20	BRCA1	NM_007294.4	c.5030_5033 delCTAA	NP_009225.1:p.Thr1677Ilefs*2	16	Frameshift	Pathogenic	rs80357580 / 37623	NGS
C21	BRCA1	NM_007294.4	c.5209A>T	NP_009225.1:p.Arg173Ter	19	Nonsense	Pathogenic	rs80357496 / 55458	NGS
C22	BRCA1	NM_007294.3	c.5266dupC	NP_009228.2:p.Gln1756Profs	19	Frameshift	Pathogenic	rs80357906 / 17677	NGS
C23	BRCA1	NM_007294.3	c.5266dupC	NP_009228.2:p.Gln1756Profs	19	Frameshift	Pathogenic	rs80357906 / 17677	NGS
C24	BRCA1	NM_007294.3	c.5266dupC	NP_009228.2:p.Gln1756Profs	19	Frameshift	Pathogenic	rs80357906 / 17677	NGS
C25	BRCA1	NM_007294.3	c.5266dupC	NP_009228.2:p.Gln1756Profs	19	Frameshift	Pathogenic	rs80357906 / 17677	NGS
C26	BRCA1	NM_007294.3	c.5266dupC	NP_009228.2:p.Gln1756Profs	19	Frameshift	Pathogenic	rs80357906 / 17677	NGS
C27	BRCA1	NM_007294.3	c.5266dupC	NP_009228.2:p.Gln1756Profs	19	Frameshift	Pathogenic	rs80357906 / 17677	NGS
C28	BRCA1	NM_007294.3	c.5266dupC	NP_009228.2:p.Gln1756Profs	19	Frameshift	Pathogenic	rs80357906 / 17677	NGS
C29	BRCA1	NM_007294.3	c.5266dupC	NP_009228.2:p.Gln1756Profs	19	Frameshift	Pathogenic	rs80357906 / 17677	NGS
C30	BRCA1	NM_007294.3	c.5266dupC	NP_009228.2:p.Gln1756Profs	19	Frameshift	Pathogenic	rs80357906 / 17677	NGS
C31	BRCA1	NM_007294.3	c.5266dupC	NP_009228.2:p.Gln1756Profs	19	Frameshift	Pathogenic	rs80357906 / 17677	NGS
C32	BRCA1	NM_007294.3	c.5266dupC	NP_009228.2:p.Gln1756Profs	19	Frameshift	Pathogenic	rs80357906 / 17677	NGS
C33	BRCA1	NM_007294.3	c.5266dupC	NP_009228.2:p.Gln1756Profs	19	Frameshift	Pathogenic	rs80357906 / 17677	NGS
C34	BRCA1	NM_007294.3	c.5266dupC	NP_009228.2:p.Gln1756Profs	19	Frameshift	Pathogenic	rs80357906 / 17677	NGS
C35	BRCA1	NM_007294.3	c.5266dupC	NP_009228.2:p.Gln1756Profs	19	Frameshift	Pathogenic	rs80357906 / 17677	NGS
C36	BRCA1	NM_007294.4	c.5434C>G	NP_009225.1:p.Pro1812Ala	22	Missense	Pathogenic	rs1800751 / 37670	NGS
C37	BRCA1	NM_007294.4	c.5467G>A	NP_009225.1:p.Ala1823Thr	22	Missense	Pathogenic	rs80357212 / 55588	NGS
C38	BRCA1	NM_007294.4	c.843_846delCTCA	NP_009225.1:p.Ser282Tyrfs*15	10	Frameshift	Pathogenic	rs80357919 / 17683	NGS
C39	BRCA2	NM_000059.4	c.1773_1776delTTAT	NP_000050.3:p.Ile591Metfs*22	10	Frameshift	Pathogenic	rs80359304 / 51190	NGS
C40	BRCA2	NM_000059.4	c.22_23delAG	NP_000050.3:p.Arg8Alafs*5	2	Frameshift	Pathogenic	rs397507623 / 51267	NGS
C41	BRCA2	NM_000059.4	c.4284dupT	NP_000050.3:p.Gln1429Serfs*9	11	Frameshift	Pathogenic	rs80359439 / 37892	NGS
C42	BRCA2	NM_000059.4	c.469A>T	NP_000050.3:p.Lys157Ter	5	Nonsense	Pathogenic	rs1593886887 / 694628	NGS
C43	BRCA2	NM_000059.4	c.5351dupA	NP_000050.3:p.Asn1784LysfsTer3	11	Frameshift	Pathogenic	rs80359507 / 37960	NGS
C44	BRCA2	NM_000059.4	c.537dupT	NP_000050.3:p.Ile180Tyrfs*3	7	Frameshift	Pathogenic	rs1566219199 / 584463	NGS
C45	BRCA2	NM_000059.4	c.537dupT	NP_000050.3:p.Ile180Tyrfs*3	7	Frameshift	Pathogenic	rs1566219199 / 584463	NGS
C46	BRCA2	NM_000059.4	c.5576_5579delTTAA	NP_000050.3:p.Ile1859Lysfs*3	11	Frameshift	Pathogenic	rs80359520 / 37975	NGS
C47	BRCA2	NM_000059.4	c.5992C>T	NP_000050.3:p.Gln1998Ter	11	Nonsense	Pathogenic	rs397507819 / 51983	NGS
C48	BRCA2	NM_000059.4	c.6010G>T	NP_000050.3:p.Glu2004Ter	11	Nonsense	Pathogenic	rs2137523213 / 2431305	NGS
C49	BRCA2	NM_000059.4	c.6468_6469delTC	NP_000050.3:p.Gln2157IlefsTer18	11	Frameshift	Pathogenic	rs80359596 / 38047	NGS
C50	BRCA2	NM_000059.4	c.6486_6489delACAA	NP_000050.3: p.Lys2162Asnfs*5	11	Frameshift	Pathogenic	rs80359598 / 38048	NGS
C51	BRCA2	NM_000059.4	c.67+1G>A	NA	Intron 2	Splicing	Pathogenic	rs81002796 / 52160	NGS
C52	BRCA2	NM_000059.4	c.67+1G>A	NA	Intron 2	Splicing	Pathogenic	rs81002796 / 52160	NGS
C53	BRCA2	NM_000059.4	c.67+1G>A	NA	Intron 2	Splicing	Pathogenic	rs81002796 / 52160	NGS
C54	BRCA2	NM_000059.4	c.67+1G>A	NA	Intron 2	Splicing	Pathogenic	rs81002796 / 52160	NGS
C55	BRCA2	NM_000059.4	c.7007G>A	NP_000050.3:p.Arg2336His	13	Missense	Pathogenic	rs28897743 / 38077	NGS
C56	BRCA2	NM_000059.4	c.721A>T	NP_000050.3:p.Lys241Ter	9	Nonsense	Pathogenic	rs876659100 / 231337	NGS
C57	BRCA2	NM_000059.4	c.7689delC	NP_000050.3:p.His2563fs	16	Frameshift	Pathogenic	rs80359674 / 52388	NGS
C58	BRCA2	NM_000059.4	c.8023A>G	NP_000050.3:p.Ile2675Val	18	Missense	Likely Pathogenic	rs397507954 / 52475	NGS
C59	BRCA2	NM_000059.4	c.8833C>T	NP_000050.3:p.Gln2945Ter	22	Nonsense	Pathogenic	rs2137619671 / 3075859	NGS
C60	BRCA2	NM_000059.4	c.8945_8946delAA	NP_000050.3:p.Lys2982Argfs*35	22	Frameshift	Pathogenic	rs80359733 / 462507	NGS

Carrier No.	Gene	Transcript	HGVS c.	HGVS p.	Exon / location	Variant type	ACMG classification	dbSNP / ClinVar ID	Method
C61	BRCA2	NM_000059.4	c.9097dupA	NP_000050.2:p.Thr3033AsnfsTer11	23	Frameshift	Pathogenic	rs397507419 / 38208	NGS
C62	BRCA2	NM_000059.4	c.9097dupA	NP_000050.2:p.Thr3033AsnfsTer11	23	Frameshift	Pathogenic	rs397507419 / 38208	NGS
C63	BRCA2	NM_000059.4	c.9097dupA	NP_000050.2:p.Thr3033AsnfsTer11	23	Frameshift	Pathogenic	rs397507419 / 38208	NGS
C64	BRCA2	NM_000059.4	c.9097dupA	NP_000050.2:p.Thr3033AsnfsTer11	23	Frameshift	Pathogenic	rs397507419 / 38208	NGS
C65	BRCA2	NM_000059.4	c.9097dupA	NP_000050.2:p.Thr3033AsnfsTer11	23	Frameshift	Pathogenic	rs397507419 / 38208	NGS
C66	BRCA2	NM_000059.4	c.9317G>A	NP_000050.3:p.Trp3106Ter	25	Nonsense	Pathogenic	rs80359205 / 52814	NGS
C67	BRCA2	NM_000059.4	c.9682delA	NP_000050.3p.Ser3228Valfs*21	27	Frameshift	Pathogenic	rs398122618 / 91530	NGS
C68	BRCA2	NM_000059.4	c.9682delA	NP_000050.3:p.Ser3228Valfs*21	27	Frameshift	Pathogenic	rs398122618 / 91530	NGS
C69	BRCA2	NG_012772.1	g.32036_48841delinsTAG	NP_000050.3:p.Gly2281ValfsX46	Ex.12-16del	NA	Pathogenic	NA	MLPA

Abbreviations: ACMG, American College of Medical Genetics and Genomics; HGVS, Human Genome Variation Society; NGS, next-generation sequencing; NA, not available. Only records with available variant-level information explicitly classified as pathogenic or likely pathogenic in the supplied variant-classification dataset are shown. Recurrent variants are presented separately for each carrier; therefore, identical variants may appear in more than one row.

Footnotes: Detailed variant-level information was available for 69 of the 79 germline BRCA1/2 carriers. For the remaining 10 carriers, the original detailed molecular reports could not be retrieved from the archived records; therefore, these cases are not included in the variant-level table. Recurrent variants are presented separately for each carrier. Patient names and institutional identifiers have been removed.
